# Uptake of Encapsulated Ferrous Fumarate Double Fortified Salt in the Public Distribution System in India: A Value Chain Analysis

**DOI:** 10.9745/GHSP-D-20-00448

**Published:** 2021-12-31

**Authors:** Meena Haribhau Jadhav, Marthi Gurunath Venkatesh Mannar

**Affiliations:** a Independent consultant, Global Health and Nutrition, New Delhi, India.; b Centre for Global Engineering, University of Toronto, Toronto, Canada.

## Abstract

Food fortification is a powerful strategy to reach large populations with multiple micronutrients added to a single food vehicle. The impact depends on the sustained provision and utilization of adequately fortified food by a large population (mainly in low-income and food-insecure settings). We apply a value chain (VC) analysis framework to diagnose and address the barriers to the uptake of encapsulated ferrous fumarate double fortified salt (DFS) distributed through public-sector-led DFS interventions in India.

We adapt the VC requirements framework proposed by Henson and Humphrey to identify and categorize barriers along the DFS VC as technological, market-related, and policy-related. We conducted a desk review of published and unpublished literature on DFS and information available in the public domain, semi-structured interviews with VC stakeholders from the private sector, program data from implementing organizations, and participation in multistakeholder consultations on DFS.

Major supply-side barriers were under-developed private markets, inconsistent demand from public markets, unpredictable returns-on-investments, and inadequate business incentives to invest in DFS. The product's weak consumer orientation, uncreated consumer demand, low awareness of fortified foods, inadequate nutrition signaling were significant demand-side barriers. Technological barriers related to the requirement of high-grade salt for DFS production and residual organoleptic property of mild discoloration of food. Policy barriers related to inadequate and irregular financing for distributing subsidized DFS through the public distribution system; insufficient policy support for risk-sharing and managing costs associated with fortification; and a weak institutional environment for sustaining DFS interventions.

Building an enabling institutional environment, demand creation through consumer awareness, strengthening institutional markets through public financing, managing cost and risks through public-private partnerships, and assuring quality during commercial scale-up are critical interventions necessary to ensure impact at scale.

## Abstract (precis)

Initiating and sustaining large-scale encapsulated ferrous fumarate double fortified salt interventions in the public distribution system in India poses several challenges that can be minimized by strengthening double fortified salt value chains.

## Teaser

**Key Findings:**

- Double fortified salt (DFS) interventions using encapsulated ferrous fumarate premix passed rigorous efficacy evaluations but faced challenges in showing impact at scale. Several technological, market-related, and policy barriers contributed to the reduced coverage and impact of the public-sector-led DFS interventions in India.
- Value chain analysis can be a useful method to identify and address demand and supply-side barriers to scaling up DFS in settings where the scale-up criteria are met.

**Key Implications:**

- Initiating and sustaining large-scale encapsulated ferrous fumarate DFS interventions in the public distribution system in India involve several challenges that can be minimized through strengthening DFS value chains. Critical interventions for addressing barriers in India's DFS value chain include— building an enabling institutional environment, demand creation through consumer awareness, strengthening institutional markets through public financing, managing cost and risks through public-private partnerships, and assuring quality during commercial scale-up.
- Considering the high price of DFS when introduced through private markets and the preference of a significant population to low-cost salt options, routing DFS through the public distribution system targeting low-income populations (that enables cost reduction through bulk purchase and subsidizes the product's retail price) is essential.

## INTRODUCTION

This article focuses on the University of Toronto double fortified salt (DFS) formulation (iodized salt fortified with iron) that uses the microencapsulated ferrous fumarate DFS (EFF-DFS) technology. While evidence on the effectiveness of DFS to reduce anemia in large-scale applications is still evolving, the efficacy of DFS has been proved.^1^^–^^3^ After a successful launch of DFS interventions in several states, the uptake of DFS in India has been slower than expected and has met several challenges.^4^ The purpose of this study is to diagnose and address the barriers that restrict the uptake of DFS through the public distribution systems in India. The intention is not to advocate for DFS but review its application through public distribution systems in India and propose solutions that can strengthen existing DFS value chains.

Micronutrient deficiencies affect nearly one-third of the global population^5^ and transcend generations, severely limiting individual potential and national development. Iron deficiency is one of the major problems of public health concern worldwide, with adverse consequences related to maternal and child health, cognitive development in children, and work productivity in adults. At least 25% and 37% of the anemia burden in preschool children and women of reproductive age, respectively, is associated with iron deficiency^6^ and is mainly a result of dietary deficiency.

Pharmaceutical supplementation programs have shown limited impact as they are often resource-intensive and face challenges related to product availability, the functioning of supply chains, cultural acceptability, coverage, compliance, and sustainability.^7^ Food-based approaches promoting foods naturally rich in micronutrients or enriched through fortification are viable, cost-effective, and sustainable solutions to micronutrient malnutrition, especially in developing countries.^7^

Food fortification, in particular, can reach large populations with multiple micronutrients added to a single food vehicle that is consumed widely and consistently. The World Health Organization included the reduction of anemia prevalence in women of reproductive age group by 50%, as one of the global targets for improving maternal, infant, and young child nutrition, to be achieved by 2025.^8^ Providing iron through large-scale fortification—along with addressing other determinants of micronutrient malnutrition (such as sanitation and health care)—is an essential intervention to achieve this global nutrition target. Sustained provision and utilization of adequately fortified food, reaching a wider population (especially those affected by micronutrient deficient diets), is critical to improving micronutrient intakes through food fortification. Salt is a universally available and affordable commodity, consumed daily in almost uniform quantities in all parts of the world. It is centrally processed and packaged, and hence an ideal food vehicle for fortification. The Universal Salt Iodization program, with mandatory salt iodization now covering 129 countries,^9^ have established the potential of salt as a global carrier of micronutrients on a continuous and sustained basis—a unique advantage over most other food vehicles.

Building on these efforts, attempts to include iron and other micronutrients along with iodine to salt are being tested and scaled up in India.^10^ Along with DFS, variations of multiple fortified salts have been developed at the University of Toronto research laboratory as a potentially viable strategy to alleviate other nutritional deficiencies such as folic acid, vitamin B-12, and zinc (Levente Diosady, University of Toronto, personal communication, June 2020).

We apply a VC framework to diagnose and address the barriers along the DFS value chain that restricted the uptake of DFS in India.

This study reviews the application of DFS to inform the design and implementation of fortified salt formulations in the Indian context. We apply a value chain (VC) framework to diagnose and address the supply- and demand-side barriers along the DFS value chain (DFS-VC) that restricted the uptake of DFS in the Indian context, specifically focusing on the public-sector-led interventions.

A VC is defined as^11^:

*the full range of activities required to bring a service from conception, through different phases of production, delivery to final consumers, and final disposal after use.*

Food VC approaches have been mainly used to improve the livelihoods of food producers, streamline the supply chain, and maximize profits,^12^ and in rural development to enhance commercial relations for economic benefits.^13^ Value chain analysis (VCA) has been used to investigate food production, nutrition, and food safety in the dairy VC in Tanzania,^14^ reduce micronutrient deficiencies while creating livelihood opportunities for VC actors through the introduction of fortified rice in Myanmar,^15^ assess the potential of the private sector manufacturers of fortified foods to reach poor households,^16^ and evaluate the status of flour fortification in Kenya on the adoption of mandatory fortification practices by millers.^17^ Thus, in the nutritional context, VCA is used as a diagnostic tool for food-based VCs to identify barriers (and potential solutions) to improved availability, accessibility, affordability, and acceptability of micronutrient-dense foods.^18^ VC-based solutions include a broad range of interventions such as information and education, research and technology, chain reorganization, and financial incentives to develop new policies and standards—used singly or in a combination.^18^

## METHODS

### Methods and Analytical Framework for the DFS-VCA

Applying the VCA method^19^ (Box) calls for different approaches depending on the VCA context and objectives. The narrow perspective of VCA is limited to the activities of a single firm and its position in relation to its suppliers and buyers. In its broad perspective, VCA looks at the complex range of activities of VC actors, moving from the production of raw materials along the linkages with other enterprises engaged in processing, trading, retailing, etc., to its final link with the consumers.^20^ Both the narrow and broader approach can sometimes transcend geographical boundaries (global VCs) depending on the location of value-chain stakeholders, especially retailers and buyers. We use the broad approach of VCA throughout this article.

BOX **Value Chain Analysis Framework**

1. Systematically map the core processes involved in taking a raw material through various stages of production, storage, and distribution, to the final consumption of the end products.
2. Map actors along the VC (e.g., producers, distributors, retailers, and consumers) and assessing their relationships in terms of the flow of products and information, profits and cost structures, destination, and volumes of domestic and foreign sales.
3. Identify the distribution of benefits along the VC by analyzing the margins and profits.
4. Assess the VC governance:
  - Internal: relationships and coordination mechanisms between VC stakeholders such as setting criteria for other actors like quality standards, delivery times, and volumes
  - External: institutional arrangements, legislation, and regulations
5. Examine the potential for upgrading at all stages along the VC through constraints and opportunities analysis.

Abbreviation: VC, value chain.

Through this exploratory study, we identify barriers (and potential solutions) to the uptake of DFS in the Indian context. We adopt a qualitative approach using the VCA as a tool for data collection and as a framework for analysis. Through VCA mapping, we begin by outlining the DFS-VC, its core functional components and processes, key stakeholders (actors), and their roles. Next, applying the “requirements of the VC for nutrition interventions” framework,^21^ we identify barriers to the DFS-VC functioning and development in India. Finally, we review the literature that addresses barriers and solutions to strengthen the VCs in the nutritional context and inform the development and strengthening of the DFS-VC in India.

Data sources include a desk review of policy documents and information available in the public domain on DFS; consumer data from sensory trials; program data from a DFS field intervention in Uttar Pradesh, India; and field implementation experiences of organizations implementing DFS interventions. All sales data and price data used in this report are from manufacturers' records, as shared by their representatives. The first author interviewed 1 premix producer and 3 DFS producers to collect primary data on DFS production and sales. Two more salt producers were interviewed briefly, but we could not get in-depth data from these sites. Limiting the field visits to large-scale manufacturers supplying DFS to ongoing public sector interventions, we visited the DFS production plants located in the major salt-producing states of Gujarat, Rajasthan, Tamil Nadu, and the premix production plant in Rajasthan. The information collected through these semi-structured interviews was further enriched and validated by documenting stakeholder perceptions in 3 national-level DFS consultations attended by the first author. The consultations involved representatives from the salt industry, organizations implementing DFS interventions, and government representatives. Data were analyzed qualitatively using thematic analysis based on the themes of the VC requirements framework.^21^ The Supplement Table presents the data collection and analysis framework.

VCA methodology has been applied for assessing nutrition VCs using different frameworks.^21^^–^^24^ Barriers in VCs of other commodities relate to consumer taste preferences and perceptions of nutritional value, regulatory failures, and institutional constraints,^25^ inadequate VC coordination, and weak producer organization,^26^ low and uncreated demand, barriers to the managing of costs, risks, and uncertainty for businesses, poorly developed markets and resulting low availability, and requirement of long-term funding for public distribution.^27^ Thus, VC barriers span the full spectrum of the demand-supply continuum and require a comprehensive framework for evaluation. To identify the barriers to the development and functioning of the DFS-VCA in India, we adopt a comprehensive framework including both demand- and supply-side requirements for well-functioning VCs.^21^ The absence of these requirements can be viewed as a barrier to the efficient functioning of the VC. The critical requirements are extracted from sources (Table 1).^21^^,^^28^

To identify the barriers to the development and functioning of the DFS-VCA in India, we adopt a comprehensive framework including both demand- and supply-side requirements for well-functioning VCs.

**TABLE 1.** Demand- and Supply-Side Requirements for a Well-Functioning Value Chain for Double Fortified Salt

Requirement	Description
Nutrition awareness	Consumers will value nutrient-dense foods only if they are aware of the benefits of improved nutrition
Signaling	Consumers should have the knowledge and skills to identify nutrient-dense foods from those that are not
Availability	Nutrient-dense foods should be available in locations that are accessible and socially acceptable to the consumers
Affordability	Consumers should be able and willing to pay for the added cost of fortification
Acceptability	Nutrient-dense foods should be acceptable to consumers based on physical appearance, organoleptic properties, consumption patterns, and preparation practices
Capturing value	Actors along the VC should be able to capture a sufficient share of the value they create through their contributions to the production processes of nutrient-dense food
Sufficient incentives along the VC	The value captured along the VC should be equitably distributed to actors in the form of incentives
Value coordination and governance	The VC stakeholders are individual entities that are interdependent and therefore require efficient coordination in sharing of information, alignment of business strategies, and implementation of joint promotional activities
Managing costs, risks, and uncertainty	The added costs and risks for adding additional nutrients to foods requires public policies that promote and sustain partnerships between the private business and the public sector
Appropriate institutional environment^28^	Adequate institutional mechanisms are required, such as legal frameworks that shape markets, food standards and regulations, and policies for managing costs and risks

Abbreviation: VC, value chain.

To make our assessment more action-oriented, we further elaborate the framework to categorize the barriers as related to technology, market, and policy.

## FINDINGS

### Mapping the Core Components, Processes, and Stakeholders of the DFS-VC

**FIGURE 1:**
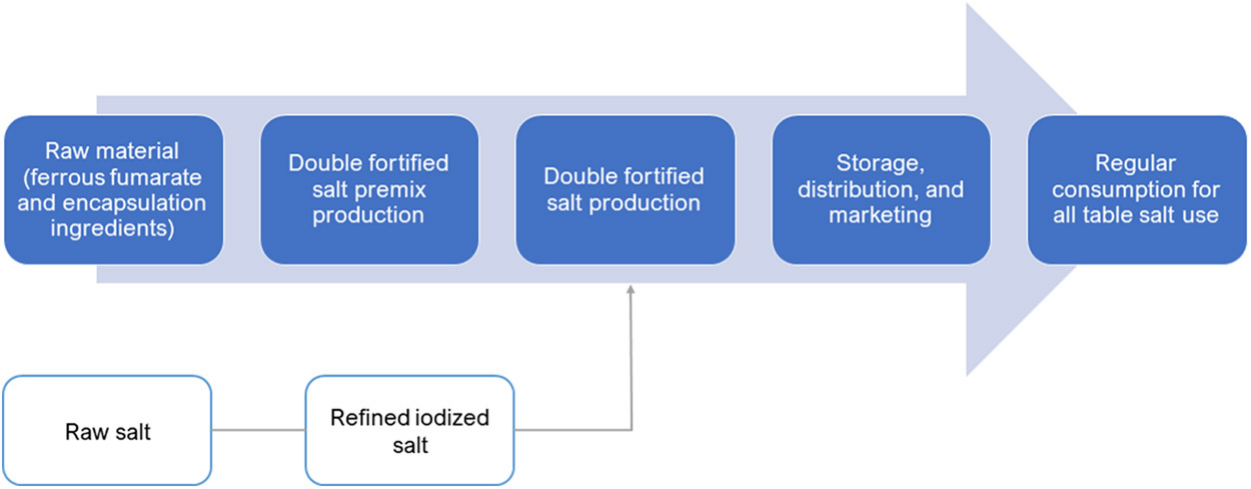
Simplified Representation of the Double Fortified Salt Value Chain in India

The core components of the DFS-VC are schematically mapped in Figure 1 and further elaborated here.

#### Raw Materials Used in EFF-DFS Production (Iodized Salt and DFS Premix)

India ranks third in salt production behind the United States and China, producing approximately 28 million of the 280 million tons of annual global salt production. Of the 28 million tons produced annually in India, human consumption accounts for 6 million tons, mostly as iodized salt. Three states: Gujarat, Rajasthan, and Tamil Nadu, produce respectively 70%, 18%, and 11% of the iodized salt produced in the country. The private sector contributes to more than 95% of salt production. The Bureau of Indian Standards sets production standards for iodized salt and DFS. The specifications for EFF-DFS production require a higher salt purity (when compared to iodized salt) of ≥98% sodium chloride, lower moisture (<1.5%), and magnesium contents (<0.1%).^29^

*DFS is prepared by adding the EFF-DFS premix to refined iodized salt. The premix consists of a ferrous fumarate iron core that is color-coated using food-grade titanium dioxide and encapsulated using hydroxypropyl methylcellulose and hydrogenated soy oil to form an encapsulated ferrous fumarate premix. Ferrous fumarate is the common form of iron used in multi-vitamin-mineral tablets. It is a recommended formulation for salt fortification based on organoleptic and bioavailability considerations. However, it is dark brownish and needs to be color masked before encapsulation and blending with salt for addition to foods. Titanium dioxide is an [U.S. Food and Drug Administration] -approved food-grade whitener, which is used in EFF-premix production to mask the dark brownish color of ferrous fumarate and make a white premix particle for blending into salt — Levente Diosady, University of Toronto, Personal communication, June 2020*

This premix is added to iodized salt at a 1:160–200 ratio to produce DFS with iron content at the specified level (850–1100 ppm).^30^^,^^31^

#### DFS Premix and DFS Production

Three DFS premix production plants in India—located in 3 states: Rajasthan, Gujarat, and Maharashtra—use the EFF technology. These plants supply premix on-demand to the DFS manufacturing plants that are currently operational in the country. The capital cost of building a premix production plant with a capacity of 1500 tons/year is estimated to be approximately INR 70 million (approximately US$1 million). To date, India has a DFS premix production capacity of approximately 6,000 metric tons/year (adequate to produce approximately 1 million metric tons of DFS). However, only 10%–20% of this capacity is currently used due to low market demand for DFS.

**FIGURE 2:**
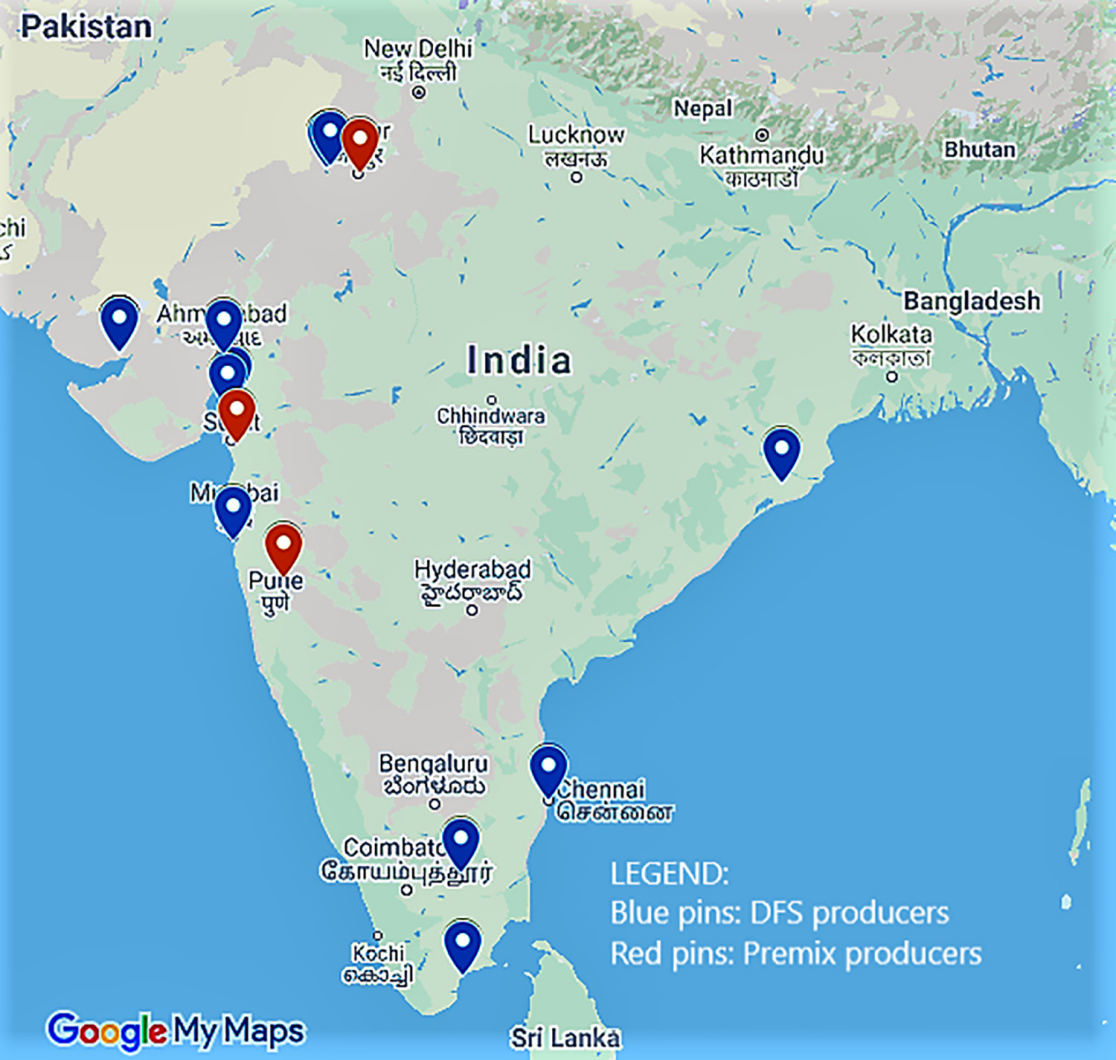
Map of Double Fortified Salt and Premix Producers in India

There are nearly 19 salt producers in India who hold a license from the Food Safety and Standards Authority of India (FSSAI); however, only 6–7 are currently operational. Figure 2 maps the number and the geographical locations of the DFS and premix producers in the country. DFS manufacturers must purchase the premix and blend it with refined iodized salt in their salt plants to produce DFS. To blend the premix with iodized salt, DFS producers require additional equipment costing approximately INR 10 million (approximately US$0.14 million). Salt fortification in India has 2 unique advantages: an established domestic salt production and distribution capacity and the concentration of more than 60% of the edible salt market across a small number of private producers (4 to 5), potentially facilitating a public-private partnership. Four years since the inclusion of DFS interventions among public health approaches to address iron deficiency anemia, the DFS market in India is still in its infancy. DFS reached approximately 4% of the country's population mainly contributed by the public-sector distribution across 6 states in 2018. Based on geographic coverage of the DFS interventions, the implementing states accounted for varying levels of population coverage ranging from (1% to 23%) **(**author's calculations) (Table 2).

**TABLE 2.** India State DFS Interventions in the Public Sector and Estimated Population Coverage^a^

State	Delivery Platform	Coverage	Quantity Procured (in MTs in 2018)	Estimated Coverage^b^ Over 1 Year (Calculated 10 g/day/person)	Estimated Population Coverage (%)	Status (January 2020)
Andhra Pradesh	ICDS, PDS	Entire state	8,080	2,213,699	4	On hold
Telangana	PDS	Entire state	NA			Ongoing
Jharkhand	PDS	Entire state	32,000	8,767,123	23	On hold
Tamil Nadu^c^	ICDS, MDM (channeled through PDS)	Entire state	2,600	712,329	1	Ongoing
Uttar Pradesh	PDS	10 districts	60,000	16,438,356	7	On hold
Madhya Pradesh	PDS	89 tribal blocks across 20 districts	50,000	13,698,630	16	Ongoing
Gujarat	ICDS	Entire state	26,500	7,260,274	11	Ongoing
India (Total)			1,79,180^d^	49,090,411	4^e^	

Abbreviations: DFS, double fortified salt; ICDS, Integrated Child Development Services Scheme; MDM, mid-day meal; MT, metric ton; NA, not applicable; PDS, public distribution system.

a Data as reported by partner organizations.

b Does not suggest actual utilization.

c Used ferrous sulfate DFS formulation.

d 3% of annual edible salt consumption of 6 million.

e Based on a population of 1.38 billion.

Four years since the inclusion of DFS interventions among public health approaches to address iron deficiency anemia, the DFS market in India is still in its infancy.

#### The Current Market and Distribution Channels of DFS

DFS distribution in India is predominantly routed through VCs of 3 major public food distribution programs with a minuscule contribution through private sector markets. Consequently, the beneficiaries of the public food distribution programs are the major consumers of DFS. The 3 major public food distribution channels operational across India are the public distribution system (PDS), the mid-day meal scheme for school children, and the Supplementary Nutrition Program for preschool-age children under the Integrated Child Development Services Scheme. The PDS (implemented through the Ministry of Consumer Affairs, Department of Food & Civil Supplies) is the key arm of India's food security system to provide essential foods to low-income populations at subsidized prices. There are more than 500,000 PDS outlets across India (also called fair price shops), serving more than 800 million beneficiaries (75% of the rural population and 50% of the urban population in the country).^32^ To date, at least 20 states procure free-flow refined iodized salt for distribution through the fair price shops. In 2016, DFS was introduced through the PDS, starting with Jharkhand and Uttar Pradesh, followed by a few other states: Andhra Pradesh, Telangana, Karnataka, and Rajasthan. Iodized salt and DFS distribution through the PDS are state-initiated interventions and not centrally funded. They vary in coverage (statewide vs. few districts, processes (prices/subsidies and quantities distributed), and sustainability (duration of government commitment to supply subsidized DFS/replace existing subsidized iodized salt).

In 2017, the Ministry of Women and Child Development, which oversees the Integrated Child Development Scheme, and the Ministry of Education, which oversees the mid-day meal scheme (the central government's 2 food distribution programs that serve hot cooked meals to preschool and school-age children) issued directives requiring the use of DFS in those schemes.^33^ Following the directive, the supply of DFS through these channels was expected to increase significantly. In public institutions such as panchayats, Integrated Child Development Scheme block offices float local DFS procurement tenders for distribution of DFS to their beneficiaries. Medium-sized retailers bid for these tenders purchase DFS from manufacturers (often rebranding the DFS with their names) and supply the Integrated Child Development Scheme and mid-day meal programs. Notwithstanding the strong political commitment and the promotion of DFS by the Government of India, the process of scaling up DFS in the public sector at the state level has been slower than expected.

DFS flows through established commercial distribution channels for iodized salt in the open market and passes through 4–5 middle-level wholesalers and retailers before it reaches retail stores for sale. Although there are currently at least 10 different brands of DFS launched in the private sector, these brands have near negligible sales and have not penetrated the commercial market. High production cost, low demand for DFS, and organoleptic changes in stored salt and cooked food have been identified as the main reasons. Nonetheless, India is the single largest producer and consumer of DFS globally,^34^ mainly due to the inclusion of DFS in the government's food security programs. The DFS-VC in India is a unique model of private production and public distribution. A total of approximately 180,000 metric tons of DFS was produced by private sector salt manufacturers and supplied to public distribution programs in 2018 (Table 2).

The DFS-VC in India is a unique model of private production and public distribution.

#### The Consumer VC of DFS

Institutional markets currently drive the demand for DFS in India. The beneficiaries of the PDS and ICDS are the primary consumers of DFS in the country (estimated DFS coverage under these schemes in 2018 at 50 million of the approximately 750 million active beneficiaries in India). They constitute a large population, mostly low-income, primarily concentrated in the rural areas, with often low awareness on nutrition and even lower on fortification, and a preference for low-cost products for daily household needs. These populations rely more heavily on purchasing essential foods at subsidized prices through the PDS. However, these populations also have cultural habits related to salt usage, such as a preference for the crystal salt and family traditions of buying specific salt brands. They also have expectations of the taste and appearance of cooked food that DFS formulations have to meet (research experiences of the author).

#### VC Governance and Policy Environment

VC governance can be categorized at 2 levels: (1) Internal, coordination of the VC stakeholders directly involved in the production and supply of the fortified commodity; (2) External, legislative, and governmental policies that govern the functioning of the VC.^20^ The internal governance mechanism of the DFS-VC in India is currently fragmented and unorganized. At the external level, the FSSAI under the Ministry of Health is the central regulating and monitoring agency for food products, including DFS. It has set standards for nutrient levels and permitted stabilizers/coating agents in DFS; standards of DFS quality, packing, and labeling of DFS packets; and standards for quality assurance at DFS factory premises.^35^ FSSAI also houses the Food Fortification Resource Centre, which acts as a program management unit for the FSSAI to inform, promote, manage, and coordinate activities related to fortified food products. Table 3 lists the key stakeholders involved in the DFS-VC and their functions that contribute to the VC.

**TABLE 3.** Stakeholders of the DFS VC in India and Their Roles

Component of DFS VC	Organizational Set-up/Stakeholders	Process/Function	End Product/Service
Production of raw material	Manufacturers of pharmaceutical/food grade ferrous fumarate	Production of pharma grade iron from crude iron	Ferrous fumarate powder (used in iron pharmaceutical preparations and DFS premix)
	Manufacturers of: Durum semolina wheat flour Titanium dioxide (food grade) HPMC HSO	Production and supply of raw materials required for binding and encapsulating the ferrous fumarate iron core: Durum Semolina – for binding of ferrous fumarate powder Titanium Dioxide – used as a whitening agent HSO and HPMC – used for encapsulation	Other raw materials (food grade/pharma grade) (used in the food processing/pharma industry and DFS premix production)
DFS premix production	DFS premix manufacturers	Ferrous fumarate powder binding to produce DFS premix core through a process of binding and extrusion. These particles are then coated with a whitening agent (Titanium dioxide) and encapsulated using soy stearin and HPMC coating	DFS premix
	Salt manufacturers	DFS premix is procured by salt manufacturers and blended with iodized salt to produce DFS	DFS
DFS production	Raw salt producers	Small scale raw salt producers mine salt from sea or lake brine and sell raw salt to refineries for further processing	Raw salt for processing to refineries/salt manufacturers
Storage distribution and marketing	Commercial distribution channels – vendor warehouses to wholesale retailers to local shops	DFS transported mainly by rail (from Gujarat and Rajasthan) and mainly by road from other salt-producing states	Availability of DFS in local shops and institutions
	Public channels – vendor warehouses to government warehouses and institutional networks such as PDS shops	Standard packing and logistics requirements for food products followed	
Consumption of DFS (Consumer value chain)	Beneficiaries of public food distribution programs	Consumption of food cooked with DFS or receiving subsidized DFS for use at homes	Regular use of DFS
	Consumers in the private sector	Purchase of table salt from markets and choice of salt	
Value chain governance	Salt producer and trader associations	Salt producer and trader associations: Coordination between salt producers and traders	Developing and sustaining the DFS market Promotion of DFS for consistent use at-scale
	Food Safety and Standards Authority of India	Setting of fortification standards Enforcement of standards through regulatory checks Promotion of fortified products through capacity building and training of manufacturers and promotional campaigns among consumers	
	Confederation of Indian Industry	Developing a market for DFS and other commercial coordination	

Abbreviations: DFS, double fortified salt; HPMC, hydroxypropyl methylcellulose; HSO, hydrogenated soy oil; VC, value chain.

### Barriers Along the DFS-VC

#### Technology-Related Barriers

The technology-related barriers mainly affect 3 aspects of the VC requirements: availability, affordability, and acceptability. Fortification has added costs, which the consumers often bear unless the costs are subsidized through public interventions. The premix cost is a significant addition to the price of fortified foods (estimated at 80%–90% of the incremental cost). Consumers must either be willing to pay for this incremental cost (with the precondition that they are aware of the increased nutrient content) or be provided with subsidized DFS through public funding. Another limitation of technology that affects affordability and availability is that high-quality salt is required for DFS production. Approximately 60% of salt in the Indian market falls in the high-grade category. Refining some salts to suit the technological and regulatory requirements of DFS is cost-intensive. State governments who invite proposals to supply DFS set up a competitive process based on the lowest-bidder contracting system, lowering the price to a bare minimum with minimal profit margins for the producers. This results in salt manufacturers compromising the quality of salt and premix used for DFS supplied to public programs.

Food fortification works best when it is a covert intervention and indistinguishable from the regular food items in its sensory properties. Technological limitations of the premix encapsulation and the compromised quality of encapsulation during commercial scale-up affect the product acceptability. Under well-controlled manufacturing conditions, it is possible to produce a high-quality premix that would produce DFS with minimal organoleptic changes when blended with a high-quality refined salt. However, when the quality of the premix or the raw salt is compromised (such as weak encapsulation of premix or substandard raw salt), the premix particles are more visible, and the discoloration of food (an inherent property of iron) is more intense.

Technological limitations of the premix encapsulation and the compromised quality of encapsulation during commercial scale-up affect the product acceptability.

#### Private and Public-Sector Market-Related Barriers

Market-related barriers are identified as the main constraints to the VC development of DFS. The barriers resulting in a weak DFS market affect almost all aspects of the VC requirements such as nutrition awareness; nutrition signaling; availability; affordability (on the demand side) and capturing value; sufficient investments for the VC stakeholders; VC coordination and governance; and managing costs, risk, and uncertainty (on the supply side). There is currently an uncreated demand for DFS, especially as a nutritional product. Low awareness regarding fortification is the primary reason for this uncreated demand. Mechanisms for nutritional signaling in the case of DFS are inadequate and have not reached most consumers, especially from the low-income groups. Institutional demand for DFS from public sector markets has been unpredictable and inconsistent. Bundling DFS with ration kits in the PDS did not necessarily ensure utilization at the consumer level (as consumers preferred to use their regular salt to which they were habituated). At the same time, behavior change interventions by governments were minimal.^10^ Additionally, the low market value of DFS and unpredictable returns make DFS a high-risk investment for salt and premix manufacturers. Fortification also involves an increase in the cost of production, primarily contributed by the premix. In the absence of subsidizing mechanisms, these costs are transferred to the consumers. Salt is a low-priced and inelastic commodity offering little scope for value capture by VC actors. An average family of 5 members spends less than INR 180 (US$2.40) annually on salt. Increased purchase and consumption of DFS is possible only if it replaces the iodized salt that consumers currently buy. Price-conscious consumers opt for a lower-value product of the same brand. In the absence of information on fortification and the low value given to salt as a nutritional product, lower-income households prefer to buy the low-priced options of iodized salt (several such brands are available with a sizeable rural market). The slow-moving DFS-VC due to uncreated demand for fortified foods is the main reason the VC stakeholders cannot capture sufficient value for the services they provide. Additionally, the DFS-VC is still undeveloped and fragmented, and the VC stakeholders are not organized. Also, policy support for risk-sharing between public and private sectors has been inadequate.

#### Policy Barriers

Policy barriers for DFS uptake relate to nutrition awareness, nutrition signaling; availability; affordability; acceptability; sufficient incentives along the VC; VC coordination and governance; managing costs, risks, and uncertainty; and appropriate institutional environment. Nutritional awareness and empowering consumers with signaling mechanisms are primarily public-sector functions. Such mechanisms, though existent, have been inadequate. The private sector rarely highlights this function unless directly related to commercial gains (profit margins). Salt being an indispensable component of household food purchases, there is a large rural market for low-priced salt brands that cannot be double fortified. Therefore, subsidizing the additional costs through policy initiatives is essential to reach this largely rural and low-income consumer segment with nutrient-enriched salt. However, the funding for subsidization of DFS needs to come from state-government budgets that are often stretched and are inadequate and inconsistent. Additionally, insufficient quality control mechanisms and their implementation resulting in poor quality DFS affected the acceptability of the product.

Efficient coordination between VC stakeholders is essential for well-functioning VCs. India has built adequate capacity for the primary production of DFS; however, “producer organization” and a well-coordinated VC is yet to be developed. Despite central governing authorities such as FSSAI and Confederation of Indian Industry (a nongovernmental trade association and advocacy group) (Table 3), linkages between the different actors in the VC are weak. Public policies play a vital role in managing the costs, risks, and uncertainty in VCs. Most DFS producers said DFS was a risky investment and expected public-sector leadership to develop the DFS market. The institutional environment for DFS scale-up is weak, despite the inclusion of DFS in policy documents. Even with political support, there is a lack of committed central government funding (that could flow to states to support PDS-based DFS interventions). The low consumer demand for DFS due to poor awareness further contributes to the low priority for DFS as a political instrument of change among policy makers. Perceived threats of public disapproval due to color changes of stored salt and cooked food (a fallout of past experiences of governments with previous DFS formulations without iron encapsulation) and change of state governments and bureaucrat transfers that initiated DFS interventions are some of the additional policy barriers affecting DFS roll-out and uptake.

## DISCUSSION

The current DFS-VC in India can be characterized as “low demand and inconsistent supply.”^22^ VC researchers have suggested the following interventions for VCs with these shortcomings: capacity building for primary production, producer organization, social marketing to stimulate demand, subsidies for consumption, and incentives for risk taking by producers and retailers.^22^ However, the following challenges are anticipated while pushing an innovative product such as DFS in VCs, especially through public-sector channels.

- Demand assessment is challenging for innovative products. Inflexible and inefficient supply chains could lead to shortages affecting the availability or overstocking of the product in the field affecting quality and consumer acceptability given the relatively short shelf life of DFS.
- A mass media campaign is necessary for overcoming the technological limitations of DFS, such as visible premix particles and color change of cooked food; however, behavior change takes time.
- Considering the slow uptake of DFS, governments would tend to replace the product with alternatives (where available) or divert the limited funding to other interventions.
- Consumer forums and citizen groups may perceive the pushed product as an infringement on their rights.
- Producers may find DFS a risky investment, given the uncertainty around DFS uptake and scale.

While these are valid concerns, DFS is still in the testing phase, and conclusive decisions would need program maturity. Focusing on strengthening the DFS-VC and closely monitoring and demonstrating the impacts on the nutrition status of populations would still make it a worthwhile intervention.

Successful DFS interventions would need a public health movement with cross-sectoral partnerships involving governments, producers, donors, researchers, and people representatives to build an environment where the innovation would survive and thrive. We propose the following interventions for strengthening the DFS-VC in India, in order of priority: building an enabling institutional environment, demand creation through consumer awareness, managing costs and risks through public-private partnerships, strengthening institutional markets through public financing, and assuring quality during commercial scale-up.

Successful DFS interventions would need a public health movement with cross-sectoral partnerships involving governments, producers, donors, researchers, and people representatives to build an environment where the innovation would survive and thrive.

### Building an Enabling Institutional Environment

Researchers adopting a VC approach have observed that political commitment is essential for the sustainability of food fortification interventions that are often business-driven but led by governments or donors.^36^ DFS programming and implementation in India is characterized by fragmented efforts by a couple of organizations with limited coordination at the national level. Advocacy efforts by these organizations have led to its inclusion in policy documents but without committed budgets. Initiating and sustaining DFS interventions are left to the Food and Civil Supplies department of state governments, who consider anemia reduction a health department mandate. Therefore, the leadership of the nutrition and health-related departments and national organizations at the central level backed by funding for the states is needed. However, with limited evidence on effectiveness, DFS remains an unproven product and has yet to attract the attention of public health advocates outside the fortification sector. The successful implementation of 2 well-established salt-related public health programs in India—universal salt iodization and salt reduction—promises a unique model for joint efforts and increased impact.

### Demand Creation Through Consumer Awareness

Consumer demand is the key driving factor around which the other elements, such as production capacity, distribution and supply chains, and marketing, get organized. In comparison to iodine, promotional activities around DFS have been minimal and nongovernmental organizations advocating for DFS are few. Mass media have been successfully used in India in other public health programs such as family planning, HIV prevention, and the salt iodization program. Such mass-media communication in the case of DFS is challenging due to the current perception of DFS as still an experimental product^37^ (mainly due to issues of acceptability resulting from a slight color change of food cooked with DFS, and the risk of being mistakenly perceived as promoting higher quantities of salt in the diet). Joint messaging in partnerships with salt reduction programs and the communication messages around anemia reduction in complementary interventions would help bolster the program.

### Strengthening Institutional Markets Through Public Financing

High investments required for quality DFS manufacturing increase the market price of DFS by 30%–50% higher than iodized salt. In private markets, the additional costs are passed on to the customers limiting DFS to be viewed as a premium product targeted at high-income consumers.^34^ In the public sector, this cost difference is much lower due to bulk purchasing (15%–30%). Given this evidence and the preference of a significant proportion of consumers to low-cost salt options, the public sector channeling DFS at a subsidized rate to low-income populations is essential. The market for DFS in India is currently driven and sustained by the public sector channels such as the PDS. In the absence of awareness of fortified products, and consequently, low demand for DFS, promoting and ideally having mandatory inclusion of DFS in public food distribution programs (especially the PDS with expansive coverage of nearly 70% of the country's population), along with adequate investments in behavior change interventions, is the quickest way to deliver the DFS to large low-income populations. Considering the low consumer demand, we suggest a quasi-mandatory approach, where DFS is mandatorily part of the government-subsidized food basket kit and available at all outlets. However, its purchase at the consumer level should be voluntary. Thus, ensuring the availability of DFS at purchase points near consumer homes and curbing any profit-driven promotion of DFS through the government channels. Demand-based inclusion of DFS in the PDS would create a more long-standing and assured market for DFS, leverage the currently underutilized capacity of DFS production plants, and create incentives for the private sector to further invest in expanding the DFS production.

Considering the public health importance of DFS and its potential to benefit large populations in a cost-effective manner, governments may also provide financial incentives to DFS producers until the programs achieve maturity. These incentives may include central government funding for subsidizing DFS, exemption or reduction of taxes on premix, and concessions in transportation taxes. When supplied and subsidized by the PDS, the incremental costs of DFS can be absorbed by government budgets to reach low-income and price-conscious consumers with DFS priced lower than the cost of iodized salt in the open market. Low-income populations are vital demography to which micronutrient interventions are directed. Routing subsidized DFS through the PDS targets the most vulnerable and affected populations at scale with more significant impact, simultaneously creating an assured public sector market that acts as a risk-sharing mechanism between businesses and the governments. While the financial burden of subsidies on government budgets is assumed to be significant, depending on their fiscal space, state governments have the choice to provide 1 or more items, such as edible oil, sugar, kerosene (cooking fuel), and iodized salt, at subsidized rates.

### Managing Cost and Risks Through Public-Private Partnerships

Investing in VCs of nutrient-dense foods serving low-income populations can involve significant risk and uncertainty for businesses.^21^ However, it could also offer an avenue to expand the market share of businesses that have already saturated the high-end markets.^38^ Public-private partnerships are an effective strategy for orienting food and nutrition VCs to the nutrition requirements of the low-income populations and those most affected by nutritional deficiencies. These partnerships can enable investments in new product development of fortified foods, expand the distribution network for existing fortified foods in rural areas, and influence consumer preferences to strengthen consumer demand for micronutrient-rich processed/packaged foods.^39^ In the case of DFS, the private sector has been limited to DFS production and supply to the state governments with DFS interventions, limiting their status to that of a contracted vendor. The salt industry has not been a partner to the public health movement around DFS, despite India having a corporate social responsibility mandate for large private organizations. However, it is encouraging to note that at least 6–7 salt producers have included DFS as a trial product and have actively bid for government procurement tenders. With an enabling institutional environment, private producers could invest in new business opportunities through DFS. The public-sector channels distributing DFS may benefit from the private-sector strengths in product research and branding, advertising and marketing, and efficiency in supply chains. Simultaneously the public sector could offer incentives to cover the financial risks to producers investing in a low-demand product, as elaborated in the section related to institutional strengthening through public financing.

### Assuring Quality During Commercial Scale-Up

There is a risk of quality being compromised during the commercial scale-up of an innovative product. The government plays a crucial role in providing guidelines and enforcing quality through rigorous checks and monitoring along the VC. Presently, the government has limited resources for establishing a robust monitoring mechanism. Donor commitments in this area through funding support and technical expertise to strengthen government capacity in this function are suggested.

This article is the first-ever review of DFS from a VC perspective and identifies bottlenecks to DFS uptake and opportunities for improvement along the VC. It also helps understand the trade-offs involved in scaling DFS as a public health strategy to address iron deficiency anemia. Additionally, it identifies solutions to be implemented through a VC framework, as often low-stream coverage problems need fixing further up the VC. The article contributes to the VC literature as an example of a practical application of the broad VCA approach to an important public health intervention to address a widely prevalent nutritional deficiency. Our article highlights the critical role of policy for the survival of an innovative health product in a low-demand market. It brings out the challenges of implementing a public health program that heavily relies on the private salt industry but is driven by government health mandates.

Finally, our findings are specific to the Indian context. The situation of India is unique due to the presence of a country-wide public sector delivery platform that enables cost-effective channeling of privately produced DFS to low-income consumers at accessible delivery points and a subsidized rate. Other countries may need different models to achieve the same level of efficiency and cost-effectiveness. They may need different subsidization mechanisms at the level of producers or consumers. Nonetheless, considering the scale of its programs, India would serve as a significant learning lab for effectiveness studies for DFS interventions once these programs attain maturity.

### Limitations

With the introduction of DFS in public health programs to address anemia as recently as 2016, the VC of DFS in India is still in the developmental stages. Performing a VC analysis at this early stage was challenging due to fewer stakeholders representing the salt industry and being actively engaged in DFS production, resulting in a small sample size for data collection. Therefore, a qualitative approach was adopted using mixed methods, relying heavily on secondary data (or physical records) and unconventional data collection methods such as consultation workshops. Reliance on secondary data may have affected data quality. Also, most of the insights for the DFS-VC come from the public sector markets (government-driven DFS interventions), missing out on the private domain due to the negligible or near-absent presence of DFS in the open market. The private sector perspectives outlined in the article relate to a few large-scale manufacturers supplying DFS to public sector programs. Their opinions are likely biased in favor of DFS linked to commercial considerations. However, given the fact that all the 3 DFS producers mentioned DFS as a failed product in the private sector (due to costs and color change of cooked food) rules out any biased responses from these manufacturers. Also, we missed out on the perspectives of other private salt manufacturers who attempted to launch DFS in the private sector but failed and others who never adopted DFS. Their insights are likely to inform action further to reposition the intervention more sustainably.

## CONCLUSION AND POLICY IMPLICATIONS

In conclusion, we identify market-related barriers as the main challenge for DFS uptake in India. The uncreated demand for fortified products (including fortified salt) and the resulting low market are crucial challenges. However, creating a need for a public health good where none exists is primarily considered a role of the public sector. Therefore, building an enabling institutional environment through public policies that promote the DFS market and regulate quality during commercial scale-up are crucial policy actions for sustainable DFS interventions. Government ownership and support for the production and promotion of DFS are essential for expanding and sustaining demand for the product. Long-term funding commitments by the government for distributing subsidized DFS could be the trigger to accelerate scale-up in the public sector (encouraging DFS producers to promote the product in the private sector simultaneously). However, once the programs achieve maturity, investments in DFS should be periodically reviewed to examine evidence on nutrition outcomes and cost-effectiveness. Strengthening DFS value chains is critical to realize the potential of DFS to have a large-scale impact in reducing iron-deficiency anemia in India.

## Supplementary Material

GHSP-D-20-00448-supplement.pdf

## Notes

**Peer Reviewed**

**First published online:** November 17, 2021.
