# Supplementary material for: Uptake of Encapsulated Ferrous Fumarate Double Fortified Salt in the Public Distribution System in India: A Value Chain Analysis
Source: Glob Health Sci Pract. 2021 Dec 31;9(4):832–45. doi: 10.9745/GHSP-D-20-00448 (PMC8691893; doi:10.9745/GHSP-D-20-00448)
Supplement: GHSP-D-20-00448-supplement.pdf [file GHSP-D-20-00448-supplement.pdf]

Supplement to: Jadhav M, Mannar MGV. Diagnosing and addressing barriers to the uptake of double fortified salt in India: a value chain analysis. *Glob Health Sci Pract*. 2021;9(4). https://doi.org/10.9745/GHSP-D-20-00448

Supplement Table 1. Secondary Data collection and analysis framework for the Double Fortified Salt-Value Chain Analysis

Data Sources	Stakeholders	Respondents/ Participants/ Organizations	Key themes for exploration	Value chain requirements [11] assessed
**Production and Sales records (Compiled data from representatives including reported data)**	Premix manufacturers	JVS Foods, Rajasthan	Production and Sales records (compiled data from representatives including reported data)<br>Price of DFS premix<br>Barriers in production and sales	Capturing value<br>Sufficient incentives along the VC<br>Managing costs, risks, and uncertainty
	DFS producers	Goyal salts – Rajasthan<br>Ankur Salts – Gujarat<br>Tamil Nadu Salt Corporation (TNSC) - Tamil Nadu	Production and sales of DFS<br>Prices of DFS premix and DFS<br>Barriers in production and sales	Capturing value<br>Sufficient incentives along the VC<br>Managing costs, risks, and uncertainty
**Web search**	Government organizations	DFS standards and guidelines – Food Safety and Standards Authority of India.<br>Policy directives of government departments including DFS interventions in food security programs (PDS – Public Distribution System of the Ministry of Consumer Affairs, Food and Public Distribution; ICDS – Integrated Child Development Services and MDM – Mid-Day-Meal scheme of the Ministry of Women and Child Development	Policies and guidelines on DFS<br>Quality Assurance, standards, and protocols<br>Coverage of DFS in food security programs<br>Policies promoting private sector investment in DFS	Signaling mechanisms (consumer education)<br>Availability (Coverage and sustainability of public sector interventions)<br>Affordability (Subsidization and inclusion in food security programs)<br>Value chain coordination and governance<br>Managing costs, risks, and uncertainty (risk-sharing through public-private partnerships)<br>Institutional environment (mechanisms, institutions, and policies)
**DFS stakeholder consultations**<br><br>**(3 consultations attended by the first author)**	Implementing organizations	Implementation partners with representation from FSSAI, Nutritional International, Tata Trusts, GAIN, and other implementation partners	Experiences of implementing organizations<br>Stakeholders' perceptions and attitudes to DFS<br>Experiences of consumers (as reported by implementation partners)<br>Barriers to implementation and scale-up	Availability (Coverage data)<br>Acceptability<br>Value chain coordination and governance<br>Managing costs, risks, and uncertainty (risk-sharing through public-private partnerships)

Supplement to: Jadhav M, Mannar MGV. Diagnosing and addressing barriers to the uptake of double fortified salt in India: a value chain analysis. *Glob Health Sci Pract*. 2021;9(4). https://doi.org/10.9745/GHSP-D-20-00448

			Coordination mechanisms	Institutional environment (mechanisms, institutions, and policies)
**Program data**<br><br>**Consumer acceptance and utilization of DFS from monitoring data of a DFS field intervention (household surveys involving 2000 households)**	The India Nutrition Initiative – Tata Trusts (program data)<br><br>Sensory studies by Institute of Home Economics, Delhi	Monitoring data from a field intervention on DFS in the state of Uttar Pradesh, India<br>Unpublished data from sensory trials on DFS done at the Institute of Home Economics, New Delhi, India	Implementation experiences and program monitoring data<br>Sensory trials and consumer acceptability	Acceptability (Consumer feedback and uptake of DFS)<br>Nutrition awareness
**Reports and studies**	University of Toronto, Canada	Telephonic discussion with representatives from the University of Toronto<br>Literature on the technology and salt formulation	Information on the encapsulation technology and the salt formulation	Acceptability

Supplement to: Jadhav M, Mannar MGV. Diagnosing and addressing barriers to the uptake of double fortified salt in India: a value chain analysis. *Glob Health Sci Pract*. 2021;9(4). https://doi.org/10.9745/GHSP-D-20-00448

Supplement Table 2. Primary data collection

Data collection methods	Stakeholders	Respondents/ Participants	Key themes for exploration	Value chain requirements assessed
**In-depth interviews (In-person interviews) (8)**	Premix manufacturers (In-person interview)	Technical manager - JVS Foods, Rajasthan<br>Operations Manager – JVS Foods, Rajasthan	Production and Sales<br>Flow of products from input to end-product<br>Barriers in production and sales<br>Stakeholder perspectives on investment in DFS premix production	Capturing value<br>Sufficient incentives along the VC<br>Managing costs, risks, and uncertainty
	DFS producers (In-person interview)	Owner – Ankur Chemfood Ltd. Gujarat<br>Manager – Ankur Chemfood Ltd. Gujarat<br>Owner – Goyal Salts, Rajasthan<br>Quality Manager – Jagannath Salts, Rajasthan<br>Officer – Tamil Nadu Salt Corporation (TNSC), Tamil Nadu<br>Owner – VKS Salts, Tuticorin, Tamil Nadu	Production and Sales<br>Flow of products from input to end-product<br>Barriers in production and sales<br>Stakeholder perspectives on investment in DFS production	Capturing value<br>Sufficient incentives along the VC<br>Managing costs, risks, and uncertainty
